# The Influence of Recombination on Human Genetic Diversity

**DOI:** 10.1371/journal.pgen.0020148

**Published:** 2006-09-22

**Authors:** Chris C. A Spencer, Panos Deloukas, Sarah Hunt, Jim Mullikin, Simon Myers, Bernard Silverman, Peter Donnelly, David Bentley, Gil McVean

**Affiliations:** 1 Department of Statistics, University of Oxford, Oxford, United Kingdom; 2 Wellcome Trust Sanger Institute, Hinxton, United Kingdom; 3 National Human Genome Research Institute, National Institutes of Health, Bethesda, Maryland, United States of America; 4 Broad Institute, Massachusetts Institute of Technology, Cambridge, Massachusetts, United States of America; 5 Solexa Ltd, Little Chesterford, United Kingdom; University of Southern California, United States of America

## Abstract

In humans, the rate of recombination, as measured on the megabase scale, is positively associated with the level of genetic variation, as measured at the genic scale. Despite considerable debate, it is not clear whether these factors are causally linked or, if they are, whether this is driven by the repeated action of adaptive evolution or molecular processes such as double-strand break formation and mismatch repair. We introduce three innovations to the analysis of recombination and diversity: fine-scale genetic maps estimated from genotype experiments that identify recombination hotspots at the kilobase scale, analysis of an entire human chromosome, and the use of wavelet techniques to identify correlations acting at different scales. We show that recombination influences genetic diversity only at the level of recombination hotspots. Hotspots are also associated with local increases in GC content and the relative frequency of GC-increasing mutations but have no effect on substitution rates. Broad-scale association between recombination and diversity is explained through covariance of both factors with base composition. To our knowledge, these results are the first evidence of a direct and local influence of recombination hotspots on genetic variation and the fate of individual mutations. However, that hotspots have no influence on substitution rates suggests that they are too ephemeral on an evolutionary time scale to have a strong influence on broader scale patterns of base composition and long-term molecular evolution.

## Synopsis

Patterns of genetic variation in the human genome provide a history of the evolutionary forces that have shaped our species. The role of one factor, recombination, in shaping variation is much debated. The observation is that regions of the genome with high recombination also have high levels of genetic variation, but this pattern can be interpreted as evidence for either repeated, widespread adaptive evolution or correlation through neutral factors such as base composition. To resolve this issue, the authors constructed a genetic map of human Chromosome 20 that has a resolution more than three orders in magnitude greater than previous maps. By comparing the location of recombination hotspots with patterns of genetic variation, evolution, and base composition, the authors show that recombination has only a very local influence on diversity, which suggests that molecular mechanisms, such as mismatch-associated repair or double-strand break formation, not adaptive evolution, drives the association.

## Introduction

The extent to which adaptive evolution has shaped the recent evolutionary history of humans is much debated. While polymorphism at certain genes, such as beta-globin or Duffy, is known to be associated with functional variation of selective importance, the functional importance of most DNA variation or substitution since the human-chimpanzee split is unknown. However, adaptive evolution is also expected to leave its footprint in patterns of genetic variation. In particular, selective sweeps that accompany the fixation of adaptive mutations will eliminate nearby genetic variation [1]. In regions of high recombination, the footprint is expected to be smaller because recombination moves the beneficial mutation onto different genetic backgrounds, allowing linked diversity to persist. The observed positive correlation between recombination rate and genetic diversity [2–4] therefore suggests that many loci have been the target of recent adaptive evolution.

However, genetic diversity is influenced by many factors, not just adaptive evolution. The rate at which new mutations appear in a population through mutation varies across the genome [5] and is influenced by base composition [6] (particularly the density of methylated CpG dinucleotides [7]), which in turn is correlated with the recombination rate [8]. Such indirect correlation may explain why the recombination rate also correlates with rates of substitution between human and chimpanzee [6,9,10] and between human and mouse [11]. Selection against deleterious mutations can also reduce genetic diversity indirectly through background selection [12], the effect of which is stronger in regions of low recombination. Gene density varies across the genome [13] and recombination hotspots typically occur outside genes [14]; therefore, direct selection against deleterious mutations in genes could also potentially lead to a correlation between diversity and recombination. There is also some evidence that recombination may itself be directly mutagenic [9,15,16].

There are two critical limitations in determining the nature of the association between recombination and diversity. First, previous analyses have relied on genetic maps estimated from pedigree studies [17], which typically have a resolution at the centiMorgan scale (approximately 1 to 2 Mb). However, recombination rates are known to vary at the kilobase scale, with much recombination occurring in short hotspots of 1 to 2 kb in length [18–20]. We would therefore expect direct (e.g., mutagenic) effects of recombination to be localised to recombination hotspots, yet this resolution is simply not available from existing genetic maps. The second major limitation is that different factors may have different (even conflicting) effects on diversity at different scales. For example, gene density could be positively correlated with mutation rate at broad scales because genes typically lie in GC-rich regions that have elevated mutation rates, yet at the very fine scale selective constraint will mean that genes themselves will tend to have lower diversity and divergence. Inference about the causal nature of the relationship between recombination and diversity requires analysis of large contiguous stretches of sequence from which it is possible to separate out the influence of different factors acting at different scales.

Here we introduce three innovations to analyse the relationship between recombination and diversity in humans. The first is the use of fine-scale genetic maps estimated from patterns of genetic variation, which provide a kilobase-scale resolution to the location of recombination hotspots [14,21]. The second is the analysis of a large contiguous region of the genome, Chromosome 20, which allows assessment of both the scale over which factors influence diversity and comparison of genic and nongenic regions [22]. Finally, we use discrete wavelet analysis [23] to assess scale-specific interactions between factors.

Informally, wavelet analysis transforms a sequence of observations (such as the GC content or recombination rate along a chromosome) into a series of coefficients that describe variation in the signal at successively broader scales. Under the simplest discrete wavelet decomposition, using the Haar wavelet function, a series of observations is essentially transformed into (1) a series of detail coefficients representing the difference between pairs of neighbouring observations and (2) a smoothed version of the original signal (note that it is conventional to rescale both the differenced and smoothed signals to preserve the variance across levels). Differencing and smoothing is repeated at successively broader scales, such that for a series of 2*^n^* observations there are *n* iterations. If multiple signals have been measured, for example, base composition, gene content, recombination rate, etc., each signal can be transformed. Correlations between signals can subsequently be assessed through linear model analysis of the detail coefficients at each level [24]. Linear model analysis of the smoothed coefficients is equivalent to assessing correlations between factors measured in windows of increasing size.

Although the transformed signal has no more or less information than the original, there are several benefits of analysing wavelet-transformed data in the analysis of genomic correlations. First, analysis of correlations at multiple scales removes the need to choose an arbitrary window size over which to search for correlations. Second, because of the way in which the transformation is constructed, the detail coefficients represent variation in the signal at a particular scale that cannot be attributed to variation at other scales (i.e., they are orthogonal to each other). Consequently, linear model analysis of the detail coefficients enables the detection of scale-specific correlations between factors. To give an illustration of why scale-specific effects can be important, note that different explanations for the link between recombination and diversity predict very different patterns with respect to the scale of the effect. If recombination is directly mutagenic we would expect to see a very local effect of recombination hotspots on diversity. In contrast, hitch-hiking explanations predict that the correlation will be over much broader scales. Finally, one useful way of thinking about linear model analysis of detail coefficients is that it measures how a *change* in one factor at a given scale influences *change* in another factor at the same scale. In effect, the analysis compares a series of paired observations and so implicitly controls for the background rate and autocorrelation of the signals. Consequently, linear model analysis of the detail coefficients is likely to be more robust to confounding factors that have not been measured. Of course, robustness may also be associated with reduced power relative to analysis of the smoothed coefficients.

**Figure 1:**
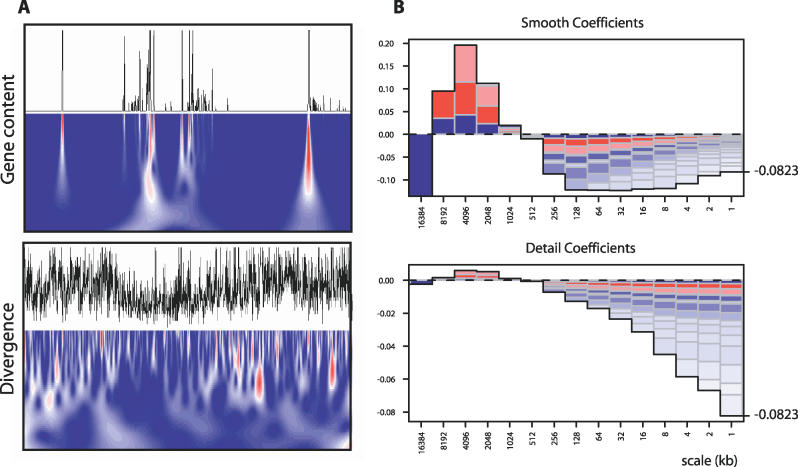
Wavelet Transformation of Genome Annotations (A) To illustrate the purpose of wavelet transformation, we show the original traces and continuous wavelet transformations using the derivative of Gaussian wavelet basis for gene content and divergence over a 2-Mb stretch of Chromosome 20. Colours indicate the magnitude (blue = low, red = high, white = zero) of the wavelet coefficients at each scale and location, with each level being normalised to have equal variance. (B) Analysis of the correlation between the smoothed and detailed coefficients at each scale (see Text S2). The height of each bar is the value of the correlation coefficient and the boxes are the contributions from broader scales (top is the broadest scale), with colour intensity related to the magnitude of the effect (blue is negative, red is positive) and size proportional to the fraction of variance explained by a given level. The correlation between divergence and constraint in the original signal (−0.0823) can be decomposed into positive contributions from correlations between detail coefficients at broad scales and negative contributions from correlations between detail coefficients at fine scales.

To illustrate these points, consider the relationship between gene content and divergence. Figure 1A shows the original signals and their wavelet decompositions over a 2-Mb region of the short arm (here a continuous wavelet decomposition is used merely for visual clarity; all analyses are carried out on discrete wavelet transformations). There is clearly both fine-scale and broad-scale variation in both signals. Correlation of the signals smoothed over successively broader scales over the long arm of Chromosome 20 (Figure 1B) shows that gene content and diversity are positively correlated when calculated in windows of 1 to 16 Mb but negatively correlated if calculated in smaller windows. Indeed, if the signals are computed in windows of 1 Mb there is no apparent correlation. Analysis of the detail coefficients explains this unusual behaviour. Over fine scales the detail coefficients show negative correlation, while at broad scales there are weak, but positive correlations. The correlation between the smoothed coefficients at any scale can be decomposed into a weighted sum of the correlations between the detailed coefficients at broader scales (see Text S2) [23]. Consequently, the detail coefficient correlations predict the behaviour of the smoothed coefficient correlations but critically also enable the separation of factors acting at different scales.

We have used wavelet analysis to assess the influences on genetic diversity along human Chromosome 20, chosen for its high degree of functional annotation [25] and availability of high-density single nucleotide polymorphism (SNP) genotype data. By combining information on patterns of diversity and divergence with information on recombination rate, base composition, and functional annotation, we show that previously reported broad-scale correlations between recombination and diversity are likely to result from indirect correlation of the neutral mutation rate with other features of genome organisation, particularly base composition. However, we also show a direct and local effect of recombination hotspots on local patterns of diversity and allele frequency, suggestive of a role for base composition biases in heteroduplex mismatch repair or double-strand break (DSB) formation. Finally, while we demonstrate highly local correlations between recombination hotspots, diversity, and GC content, we find no local correlation between recombination and divergence. These results are consistent with recent observations that while the fine-scale structure of recombination appears to evolve rapidly [26,27], rates over broader scales may be constrained [14].

## Results

We calculated summary statistics of genetic diversity (average pairwise differences between shotgun reads; see Text S1), divergence (proportion of nonidentical bases in the human-chimpanzee alignment), recombination (average recombination rate), base composition (GC content), and gene content (proportion of DNA in annotated exons) for 1-kb windows along the 62-Mb Chromosome 20 (excluding the centromere and heterochromatic regions); see Dataset S1. For each series of observations we obtained wavelet transformations using the Haar wavelet basis (similar results were obtained using other basis functions) on largest possible subsets of the short and long arm (16 Mb and 32 Mb, respectively); the two arms being analysed separately for replication. Tools for performing and analysing wavelet transformations are available within Dataset S2.

### Summarising Wavelet Transformations and Pairwise Correlations

**Figure 2:**
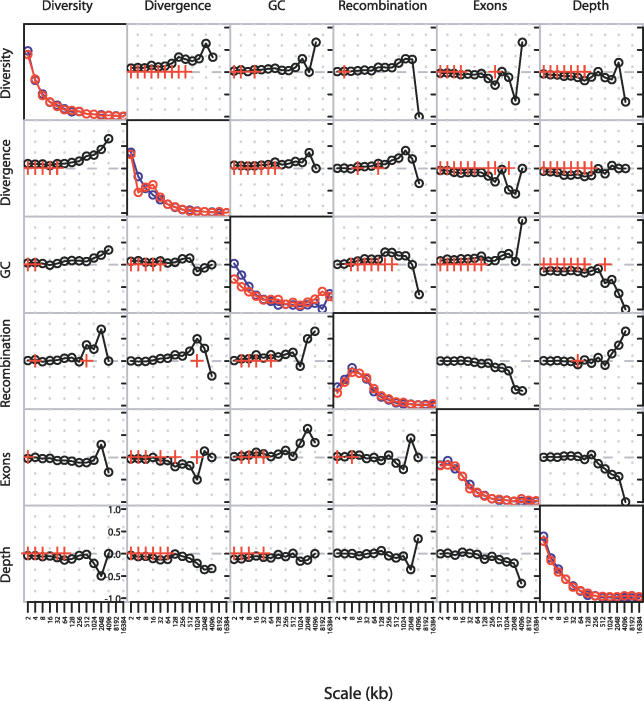
Power Spectra and Pairwise Correlations of Detail Wavelet Coefficients Diagonal plots show the power spectrum of the wavelet decomposition of each factor on the long (red) and short (blue) arms of Chromosome 20. Off-diagonal plots show the rank correlation coefficient between pairs of detail wavelet coefficients at each scale on the long (top right) and short (bottom left) arms. Red crosses indicate significant correlations (*p*-value < 0.01; Kendall's rank correlation). Scale is shown in kilobases.

A useful feature of the wavelet decomposition is that the variance in the original signal is proportional to the sum over all levels of the sum of squares of the detail coefficients at that level. Consequently, the proportions of the total variance explained by heterogeneity at different scales, known as the power spectrum of a signal, can be used to characterise the signal's distribution. Before focusing specifically on recombination and diversity, we first explored the power spectra and pairwise correlations between wavelet coefficients at each scale for all factors (Figure 2). Power spectra for each of the measured features are shown along the diagonal in Figure 2, for the short arm (blue) and the long arm (red). Broadly, we observe three different patterns. For diversity, divergence, and read depth, the greatest source of heterogeneity is at the finest scale (2 kb), and successively broader scales show successively weaker contribution. For GC content, we find a bimodal distribution, with peaks at both very fine scales (2 to 8 kb) and very broad scales (8 to 32 Mb). For recombination and, to a lesser extent, gene content, we find the greatest contribution to heterogeneity is made by intermediate scales; approximately 8 kb in the case of recombination.

Three factors influence the observed power spectrum. First, there are underlying biological factors that can determine scale-specific effects. For example, recombination hotspots are roughly 1 to 2 kb in width [18] and 50 to 100 kb apart [14], genes average about 27 kb [13], and the average read is about 500 bp [28]. Second, there are inherent limits in the resolution to which factors can be determined; for example, estimates of recombination rate are limited by SNP density in the genotype data, which is approximately 2 kb. Third, there is inherent noise in the estimation of certain quantities. For example, levels of diversity and divergence are used to estimate the polymorphism and substitution rates, respectively. However, both observations are strongly influenced by chance (which mutation or substitution events have occurred); hence, there is statistical error in the estimation of the underlying rate.

The off-diagonal elements of Figure 2 show the pairwise correlations (summarised by Kendall's rank correlation) between detail coefficients at each scale (short arm on bottom left, long arm on top right). The pairwise correlations between the smoothed coefficients are shown in Figure S1 in the supporting material. Red crosses indicate significant (*p* < 0.01) correlations. A more detailed analysis of the correlates of diversity and recombination is given below. However, there are several points worth noting from this analysis. First, diversity and divergence levels show significant positive correlation down to the finest scale (2 kb). While this is expected given variation in the neutral mutation rate, it nevertheless indicates that the level of statistical error involved in measuring these quantities at the kilobase scale is not sufficient to obscure signal. Second, levels of diversity show significant correlation with all other factors over at least some scales; recombination does not show as many significant correlations, though its positive association with GC content over many scales (4 to 512 kb) is notable. These observations reinforce the need for multiple factors to be considered in assessing correlations between recombination and diversity. Third, we find that read depth is very significantly negatively correlated with base composition, diversity, and divergence over scales of 256 kb and less. This is most likely due to the lower cloning efficiency of GC-rich regions. Because base composition also influences the types of mutation observed, it is therefore important to take read depth into account when assessing the effect of recombination on diversity in this study.

### Recombination Has a Very Local Influence on Levels of Diversity

As seen in Figure 2, recombination and diversity do show some consistent statistically significant correlations (e.g., at the 4-kb scale). To extend the analysis, we used linear modeling in which diversity, divergence, and the recombination rate were log-transformed prior to wavelet analysis. Log transformation is required; otherwise, the extreme nonnormality of the residuals violates the assumptions of the linear model analysis.

**Figure 3:**
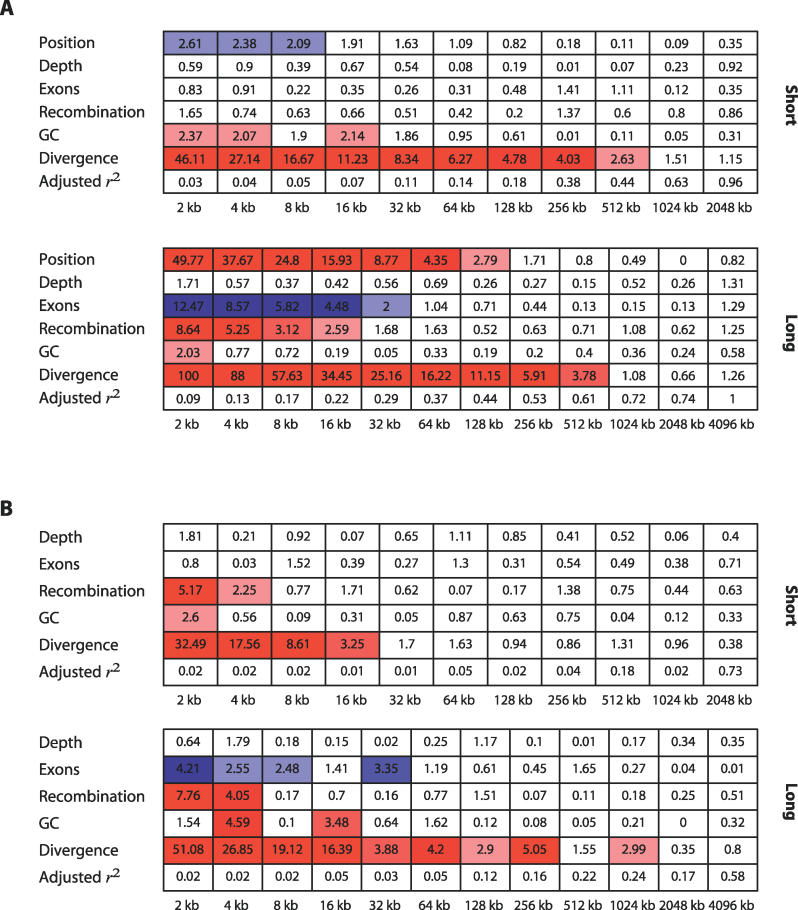
Marginal Significance (−log_10_ p-value as Determined by *t-*Test) of the Wavelet Coefficients from Four Annotations as Predictors of the Coefficients of the Decomposition of Ascertainment Panel Diversity Red boxes highlight significant positive linear relationships and blue negative. The intensity of the colour is proportional to the degree of significance. (A) Smoothed coefficients. (B) Detail coefficients. Also shown is the adjusted *r* ^2^, which can be interpreted as the proportion of the variance in the signal explained by the linear model.

First, to compare our results with previous analyses, we performed linear model analysis of the smoothed coefficients including physical position as a covariate. This is equivalent to assessing correlations between statistics averaged over different window sizes. Figure 3A shows the results of the linear model analysis of diversity on the short and long arms. Divergence is a significant positive predictor over scales up to 1 Mb, while proximity to the centromere has a suppressing effect and GC content has a weak, but consistent, effect over and above divergence at fine scales. The effects of recombination and gene content are surprisingly different between the short and long arms. On the short arm, neither factor shows a significant correlation. On the long arm, exons show a strong negative correlation up to scales of 32 kb, while recombination shows a strong positive effect over scales up to 256 kb. Gene density on the short arm is less than half that on the long arm (and there are fewer observations at a given scale); hence, the lack of association with exons is probably due to power. It is not clear why recombination shows such a marked difference.

Figure 3B shows the results of the linear model analysis on the detail coefficients (note that chromosomal location is excluded because detail coefficients are constant at a given level). There are two notable differences between the analysis of the smooth and detail coefficients. First, in contrast to the analysis of smoothed coefficients, the detail coefficient analysis shows a positive and significant effect of recombination on diversity at fine scales (2 to 4 kb) on both arms. Second, analysis of the detail coefficients shows that apart from divergence, other significant factors are only influential at scales of 32 kb and less and particularly the finest scale (2 kb). Both results suggest that the factors influencing diversity are primarily very local in nature. In particular, recombination does not exert an influence on changes in diversity beyond the scale of 2 to 4 kb; approximately the same size as recombination hotspots. To summarise the linear model analysis of the detail coefficients, we find extensive association between divergence and diversity, with additional and largely local influences of base composition, gene content, and recombination. These are likely to represent the influence of local GC content (e.g., CpG density), selective constraint, and recombination hotspots, respectively.

### Recombination Hotspots Have No Direct Effect on Patterns of Human-Chimpanzee Divergence

**Figure 4:**
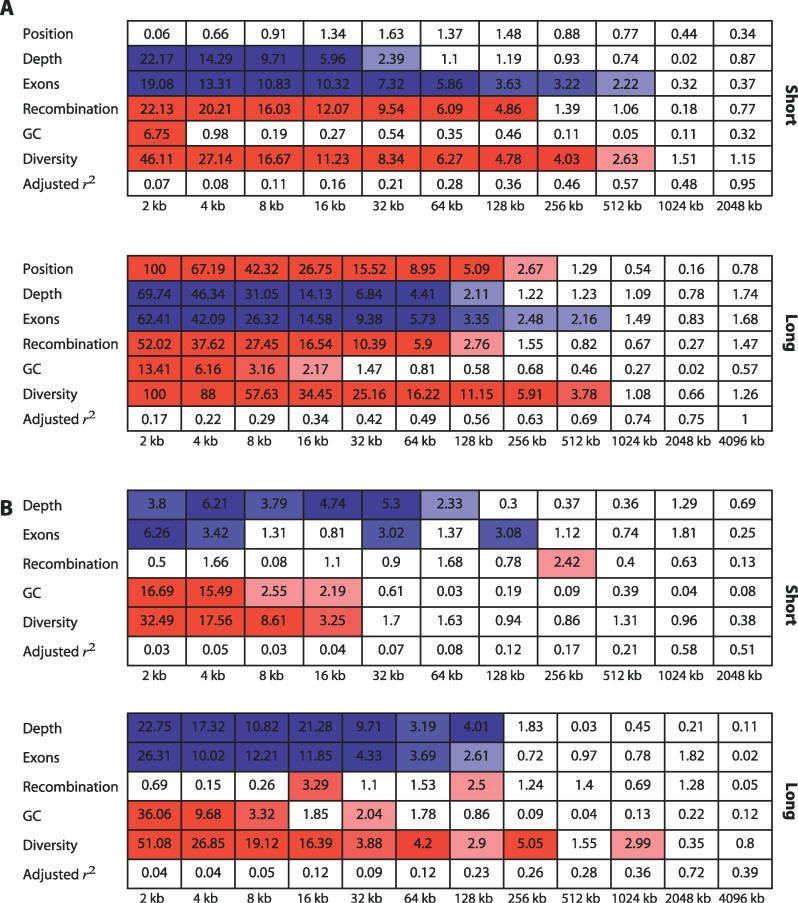
Marginal Significance (−log_10_ p-value as Determined by *t-*Test) of the Wavelet Coefficients from Four Annotations as Predictors of the Coefficients of the Decomposition of Human-Chimpanzee Divergence Red boxes highlight significant positive linear relationships, and blue boxes, negative. The intensity of the colour is proportional to the degree of significance. (A) Smoothed coefficients. (B) Detail coefficients.

Neutral explanations for a link between recombination and diversity, as is suggested by the very local nature of the association found in the analysis above, predict that if recombination influences diversity, it should also influence the rate of substitution [6]. We therefore repeated the linear model analysis, but this time with divergence as the response variable and diversity as a predictor (Figure 4).

As shown in Figure 4A, linear model analysis of the smoothed coefficients shows a strong positive relationship between recombination and divergence over scales of up to 128 kb. We also find strong associations between divergence and gene content, base composition, diversity read depth, and proximity to the centromere (on the long arm only). However, when we analyse the detail coefficients (Figure 4B), we find that read depth and gene content have significant negative effects, GC content and diversity have significant positive effects, but there is no consistent evidence for any effect of recombination. Indeed, the only suggestive relationships between divergence and recombination occur at broad scales (16, 128, and 256 kb).

Why should a factor show strong significant correlation in a linear model analysis of smoothed coefficients but not in the analysis of detail coefficients? There are three possibilities. First, the different approaches are likely to have different power to detect causal relationships. However, analysis of detail coefficients does detect significant associations when analysis of the smoothed coefficients does not, suggesting that power is not the primary difference. Furthermore, the significant effects in the detail analysis are at a broader scale, where we expect lower power (because there are fewer observations) than those in the smoothed analysis. Second, the different approaches will be differently susceptible to controlling for confounding third factors. That is, recombination and diversity could be linked through a third factor that is not included in the linear model. However, because the analysis of the detail coefficients is looking at how *changes* in one factor influence *changes* in another (effectively controlling for the background rate), rather than just whether the absolute levels are correlated, it is likely to be less susceptible to the identification of spurious correlations. Finally, there may be some biological reason for the discrepancy. For example, there is considerable evidence that humans and chimpanzees have very different recombination hotspots [26,27,29,30], indicating rapid evolution of the fine-scale structure of recombination rate variation. Consequently, a causal relationship between divergence and recombination could become “blurred” in a manner that means detail coefficient analysis can no longer identify the relationship, whereas smoothed coefficients can.

### Recombination Hotspots Have a Direct Effect on the Frequency Spectrum of Mutations

As suggested above, the very local nature of the association between recombination and diversity suggests a neutral explanation associated with recombination hotspots. Recombination could influence genetic variation directly; for example, DSBs both require DNA synthesis for repair (hence can potentially introduce copying errors) and may also expose DNA to cellular mutagens. Evidence for a mutagenic effect of recombination comes from both direct experiment [31–34] and analysis of rates of molecular evolution [6,9,11,35].

Alternatively, recombination can influence the fate of existing mutations; for example, allelic differences in the rate of DSB formation will effectively lead to meiotic drive against the more recombinogenic allele [36], and heteroduplexes formed during DSB repair (the pairing of DNA strands from homologous chromosomes) can lead to DNA base mismatches at SNPs which may be repaired in a biased manner [8,32]. Biases toward GC substitutions in regions of high recombination [37–39] (particularly high male recombination [40]) have been interpreted as evidence for the biased gene conversion (BGC) hypothesis, as has the tendency for AT→GC mutations to segregate at higher frequencies than GC→AT mutations [41,42]. Whether BGC could generate increased diversity in hotspots depends on the strength of the effect and the relative rate of appearance of GC→AT and AT→GC mutations [43]. Finally, we should also consider the possibility that estimation of recombination rates from genetic variation will tend to lead to an association between hotspots and diversity because there will be little power in low diversity regions to detect hotspots.

As described above, one approach to distinguishing between hypotheses is to consider the frequency spectrum of different types of mutations. Under the BGC model, we would expect AT→GC mutations to segregate at higher frequencies than GC→AT mutations [41], because the dynamics of BGC are, under certain assumptions, identical to the dynamics of selected mutations [44]. In contrast, a directly mutagenic effect of recombination predicts no differences between the frequency spectra for the different mutations (or even an excess of rare AT→GC mutations if hotspots and the mutations they induce are typically recent). To assess the influence of recombination on the frequency of different types of mutation, we used the chimpanzee sequence to estimate the ancestral allele for all the SNPs that had been genotyped and then considered the frequency spectrum of different classes of mutation in regions of very high and very low recombination (the top and bottom 10% of the empirical distribution, respectively).

**Figure 5:**
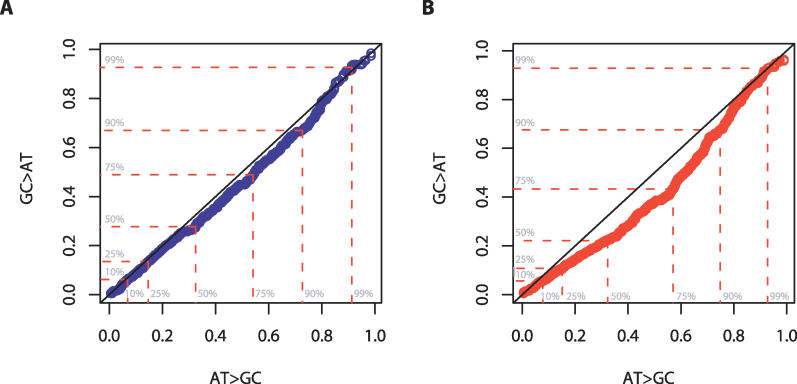
Quantile-Quantile Plots Showing the Difference in Allele Frequency Spectrum for AT→GC Mutations and GC→AT Mutations in Regions of Low and High Recombination If the two types of mutation were to have the same allele frequency distribution, we would expect to see a straight line. In both cases, AT→GC mutations are typically at higher frequencies than GC→AT mutations; however, the effect is more pronounced in regions of high recombination [(A), low recombination; (B), high recombination]. A quantification of the difference can be found in the text and supporting material.

Figure 5 shows quantile-quantile plots comparing the frequency of AT→GC to GC→AT mutations in regions of low and high recombination. In both cases we find that AT→GC segregate, on average, at higher frequencies than GC→AT mutations, but this effect is much stronger in regions of high recombination (*p* < 0.001; Wilcoxon rank sum test). This result provides strong evidence for the BGC hypothesis; however, to provide a more quantitative assessment of the effects of BGC, we modified the parametric approach of [45] (see Materials and Methods). The approach estimates the strength of BGC by fitting a population genetics model to the frequency distribution of SNPs from the African-American population (chosen because previous studies have suggested that African populations have patterns of genetic variation most compatible with a constant population size [46]) in each quintile of recombination rate (Figures S2 and S3). For SNPs in low recombination regions, the population-scaled parameter for BGC is estimated to be about 0.5, compared to approximately 1.3 in regions of very high recombination. If the effective population size is 10,000 and the “average recombination hotspot” recombines every 1,300 meioses [47], the parameter estimates for the high recombination region equates to a bias of approximately 4% toward GC at GC:AT mismatches. Note, however, that the difference in average recombination rate between low and high recombination regions is several orders of magnitude. If biases toward GC were strictly crossover dependent, we would expect to see no bias in the allele-frequency spectrum in the low recombination regions. That we see such a bias indicates either very recent changes in the mutation process (i.e., a sudden increase in the relative rate of GC→AT mutations) or unidentified base-composition biases in mutation detection.

### Recombination Hotspots Are Associated with Local Increases in GC Content

**Figure 6:**
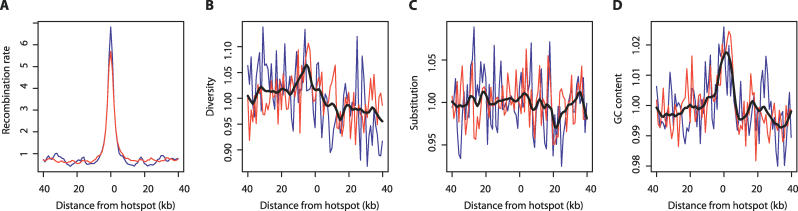
Effects of Recombination Hotspots on Genomic Features (A) The elevation of relative recombination rate around defined hotspots on the short (blue) and long (red) arms of Chromosome 20. (B) Elevation of relative diversity around hotspots (the black line is a smoothed average of the two arms). (C) There is no effect of hotspots on relative divergence. (D) Hotspots are associated with local increases in relative GC content. Note that a relative scale was used because the long and short arms can have systematic differences in absolute value.

To quantify the local effects of recombination hotspots on diversity and other factors we identified regions with very elevated recombination rate (at least 5-fold elevation over the chromosome-wide average) and plotted the average value of diversity, divergence, and base composition as a function of distance from the midpoint of such regions (Figure 6). Figure 6B shows an elevation in diversity of 6% to 7% associated with close proximity (within 10 kb) to the hotspot centre (interestingly, this seems more pronounced to the side of the hotspot rather than directly at its centre). Figure 6C shows, as described above, that there is no local increase in substitution rate associated with proximity to recombination hotspots. However, Figure 6D shows that there is a small, but repeatable increase in GC content (1% to 2%) that is again highly localised to recombination hotspots.

There are two possible explanations for the local association between recombination and GC content. Either GC-rich regions promote the occurrence of hotspots, as has been suggested in yeast [48], or hotspots, through BGC or biased mutation, cause the accumulation of GC increasing mutations. However, as discussed above, the latter explanation seems unlikely, because hotspots appear to evolve too fast to have lasting effects on base composition. Therefore, it seems most likely that hotspots preferentially occur in locally GC-rich regions. It also seems unlikely that the local association between recombination and diversity is due to the higher mutability of GC-rich regions, in particular, CpG dinucleotides [7], because we would also expect to see a locally increased rate of substitution. The data are, however, compatible with a model in which recombination is mutagenic, BGC acts on polymorphism within hotspots, and hotspots evolve rapidly.

## Discussion

Our primary conclusion is that while recombination exerts a local and direct influence on genetic variation, other factors such as base composition variation underlie the previously described broad-scale correlations between recombination and diversity in humans. Consequently, there is no need to invoke indirect effects of natural selection (such as hitch-hiking and background selection) to explain the observed correlations. This is not to say that adaptive evolution does not occur, merely that estimates of the frequency of selective sweeps or the strength of background selection [49] must take into account correlations between recombination and diversity arising from nonselective processes. As is clear from Figure 4, using divergence to control for variation in the neutral rate does not fully account for variation in levels of diversity.

### Does BGC Drive Base Composition Evolution?

While the evidence presented suggests the action of BGC, and we find evidence of very local increases in GC content around recombination hotspots, we do not believe that BGC is sufficient to drive the evolution of base composition. First, most recombination occurs in short hotspots that across the genome occupy at most 100 Mb (50,000 hotspots of 2-kb width) [14], or 3% of the genome [14]. Because BGC is intimately linked to the formation of meiotic DSBs, it will only be a strong enough force to shape base composition evolution in recombination hotspots. Second, even in regions of high recombination we estimate that the effects of BGC are equivalent to an advantageous mutation with population-scaled selection coefficient of only 1.3, when a value of 1 is usually regarded as the boundary of neutrality [50]. Third, the rapid evolution of recombination hotspots strongly limits the local effects of BGC on base composition. Combined, these factors suggest that BGC is unlikely to have a strong effect on base composition evolution, though possible factors that could increase its power include a high rate of DSBs that resolve as gene conversion events rather than crossing-over events [51] and constraints in recombination rate over larger scales [14] that will target “evolving” hotspots to particular genomic regions.

In short, while there is a strong relationship between recombination and GC content, most of the relationship is explained by scales broader than recombination hotspots (16 to 256 kb; unpublished data) and may well result from interactions of both factors with additional processes such as chromatin organisation or replication timing. Similar arguments apply to the question of whether a GC bias in recombination-associated mutation can explain the relationship between GC content and recombination.

Another important question is whether, under a plausible range of mutation biases and effective-selection coefficients, we really should not expect to see a local relationship between recombination and divergence under the BGC model given that we see one between recombination and diversity. As indicated above, it is possible that local recombination rates may evolve too fast to have any detectable influence on substitution rates [10]. Furthermore, even if recombination rates were stable, population genetics models [43] predict that increases in levels of diversity that arise from selection counteracting mutation bias are proportionally much stronger than any increase in the total rate of divergence. Nevertheless, we might expect to see local changes in the relative substitution rates of GC-increasing and GC-decreasing substitutions at recombination hotspots. Without an outgroup sequence it is not possible to determine the ancestral state of the fixed human-chimpanzee differences observed here. However, when such sequence becomes available over large regions of the genome, the prediction of the BGC model is a local excess of fixed AT→GC mutations (relative to GC→AT ones) in hotspots.

### Using Genome-Wide Surveys to Assess Evolutionary Hypotheses

We have shown that wavelet-based methods provide a powerful way of analysing scale-specific patterns in the human genome, capable of revealing novel patterns. While previous applications of wavelets in genomics [52,53] have typically considered single features, a particularly powerful use of these tools is in linear modeling, asking questions about which factors are directly correlated. Furthermore, wavelet techniques provide a natural approach to combining genomic information from multiple sources, including the raw sequence, analysis of molecular diversity and divergence, and functional annotation.

## Materials and Methods

### Genome annotations.

The location of exons and variation in base composition were obtained from the finished and annotated chromosome sequence (build 34; exon locations from Ensembl: http://www.ensembl.org). Divergence was assessed from alignments of the human and chimpanzee sequences (available from UCSC: http://genome.ucsc.edu), while diversity was assessed from multiple shotgun sequencing (SNP-discovery) projects of individuals from diverse geographic populations (see Text S1 for details). Genetic diversity was estimated from the average pairwise differences per site between aligned reads that mapped to the given window. Although average pairwise differences should provide an unbiased estimate of the population genetics parameter θ = 4*N_e_u* (where *N_e_* is the effective population size and *u* is the mutation rate per site per generation), systematic biases in sampling (for example, different samples from different populations being sequenced to different average depths) could result in a systematic association between read depth and diversity; we therefore included read depth as a covariate in all analyses. The fine-scale pattern of recombination rate variation along the chromosome was estimated from a dense genotyping survey of approximately 30,000 SNPs from individuals in three populations (96 UK Caucasians, 97 African-Americans, and 42 Asians) using a previously described coalescent-based method [19]. Recombination rates were estimated separately in each population, and the normalised genetic maps were averaged to provide a single genetic map. A gzipped, comma-delimited text file containing the features analysed in 1-kb windows is available as part of the supporting online material (Dataset S1).

### Wavelet analysis.

All genomic features were calculated for windows of 1 kb along the 62-Mb chromosome (excluding centromeric and heterochromatic regions). For each signal, we applied a discrete wavelet transformation (using the Haar base function; similar results were obtained with other wavelet bases), providing information on heterogeneity in each signal at scales of 1 kb to 16 Mb (in powers of 2). For replication, we analysed largest possible subsets of the short and long arms separately (16.4 Mb and 32.8 Mb, respectively). At such high resolution, the number of SNPs or substitutions is likely to have a considerable stochastic element. However, while sampling noise will reduce the variance we can explain through linear modeling, working at the fine-scale allows us to detect very local interactions such as may be important for detecting the effects of recombination hotspots or constraint. Furthermore, smoothing detail to coarser scales is equivalent to ignoring the fine-scale wavelet coefficients; hence, it is possible to reconstruct the variance in signal explained at any smoothed scale. Pairwise correlations between wavelet coefficients at each scale were calculated using Kendall's rank correlation, while linear model analysis was carried out at each level with the intercept forced through the origin. Scripts for wavelet analysis using packages within the R statistical computing language are available as part of the supporting material (Dataset S2).

### Frequency spectrum analysis.

The ancestral state of SNPs was inferred by parsimony from the human-chimpanzee alignments. Where no orthologous chimpanzee sequence was available, or the chimpanzee allele did not match either human allele, the SNP was excluded from further analysis. The parametric analysis of allele frequency was similar to that of [45]. Briefly, the method of [54] is adapted to account for SNP ascertainment bias, which we model as a Poisson distribution for read depth (trimmed between values of 4 and 20) and the double-hit requirement such that each SNP has to be seen at least twice. To assess how well the model fits, we first estimated (by maximum likelihood, assuming all SNPs have the same BGC value) the average read depth and strength of gene conversion (*G* = 4*N_e_ct*, where *N_e_* is the effective population size, *c* is the per-site rate of initiation of gene conversion, and *t* is the average tract length) for GC→GC, GC→AT, AT→AT, and AT→GC mutations separately (Figure S2). We estimate that

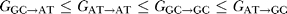
that *G*_GC→AT_ is significantly less than zero, that *G*_AT→AT_ and *G*_GC→GC_ are not significantly different from zero, and that *G*_AT→GC_ is significantly greater than zero. Comparison of the predicted frequency distribution to that observed (by visual inspection and binomial tests of the counts of alleles at each frequency) indicates that the model is a reasonable fit. We then fit a model in which *G*_GC→AT_ = −*G*_AT→GC_, dividing SNPs into quintiles of recombination rate. Estimated rates, with and without the exclusion of potential CpG mutations, are shown in Figure S3. We do not find that the exclusion of potential CpG mutations has a large or systematic effect on the estimated *G* parameter.

## Supporting Information

Dataset S1 Data Used in the Wavelet Analysis A gzipped, comma-delimited file containing details of SNP discovery and other chromosomal features in 1-kb windows along human Chromosome 20 in build 34 coordinates (hg16). Columns are window start (bp), window end (bp), number of SNPs called across window (NB, this includes redundant calls), total bases sequenced that map to window, average read depth within window, number of unique SNPs (i.e., nonredundant set), number of “double-hit” SNPs (where both alleles have been observed two or more times), number of non-N bases in the reference sequence in the window, estimate of theta, estimate of Pi, estimate of heterozygosity, percent GC, recombination fraction for window (cM), percent sequence identity between human and chimpanzee (panTro1), number of bases in window identified as being in an exon, and number of SNPs typed in genotyping study within window.
(1.8 MB TXT)

Dataset S2 R-scripts for Performing Wavelet Analyses Presented in the Paper The scripts can be used to generate some of the figures in the paper by saving both the unzipped Dataset S1 file (saved as “Chr20_1kb.csv”) and the Dataset S2 file (saved as “PLoS_code.r”) in the same folder. After starting the R program, change directory to that in which the files were saved and type source (“PLoS_code.r”) at the prompt. The functions in the scripts, however, can be used on any dataset of 2*^k^* observations. This code is powered by existing R language libraries and should be used simply as an exposition of wavelet analysis.
(15 KB TXT)

Figure S1 Power Spectra and Pairwise Correlations of Smoothed Wavelet Coefficients Diagonal plots show the power spectrum of the wavelet decomposition of each factor on the long (red) and short (blue) arms of Chromosome 20. Off-diagonal plots show the rank correlation coefficient between pairs of smoothed wavelet coefficients at each scale on the long (top right) and short (bottom left) arms. Red crosses indicate significant correlations (*p*-value <0.01; Kendall's rank correlation). Scale is shown in kilobases.
(303 KB PDF)

Figure S2 Estimates of the Strength of Gene Conversion from Allele Frequency Distributions Mutations were classified into four categories (GC→GC, GC→AT, AT→AT, and AT→GC on the basis of comparison of the human alleles with that of the Chimpanzee reference sequence). For each class we calculated the likelihood for a grid of values of the strength of gene conversion (the parameter *G* = 4*N_e_ct*) and average read depth in the SNP ascertainment panel (modeled as a trimmed Poisson distribution). The heat chart represents the likelihood surface (white is the highest likelihood), with the cross-hair showing the joint maximum likelihood estimates and the red points showing the marginal maximum likelihood estimates for *G* conditioning on values of average read depth. Contour rings marks the estimated confidence intervals (calculated by assuming twice the difference in log likelihood between models is approximately χ^2^ distributed with 2 degrees of freedom) for *p*-values of 0.05, (solid line), 0.01, 0.001, and 0.0001, respectively.
(256 KB PDF)

Figure S3 Maximum Likelihood Estimates of the Strength of Gene Conversion (*G* = 4*N_e_ct*) from SNPs in the African-American Population Sample for Each Quintile of the Recombination Rate In this analysis we assume that *G*_AT→GC_ = −*G*_GC→AT_. Estimates are shown both including (A) and excluding (B) potential CpG mutations. Also shown are estimates of *G* for SNPs in quintiles of GC content [(C) note that recombination and GC content are strongly correlated]. Solid and dashed lines indicate maximum likelihood estimates and maximum likelihood estimates conditional on a mean read depth of 13, respectively (these are largely identical).
(175 KB PDF)

Text S1 SNP Discovery and Estimation of Diversity (32 KB DOC)

Text S2 Note on the Relationship between Correlation Coefficients for Raw and Wavelet-Transformed Signals (48 KB DOC)

## Abbreviations

BGC: biased gene conversion
DSB: double-strand break
SNP: single nucleotide polymorphism
