# Supplementary material for: The Influence of Recombination on Human Genetic Diversity
Source: PLoS Genet. 2006 Sep 22;2(9):e148. doi: 10.1371/journal.pgen.0020148 (PMC1575889; doi:10.1371/journal.pgen.0020148)
Supplement: Supplementary file 1 [file pgen.0020148.sg001.pdf]

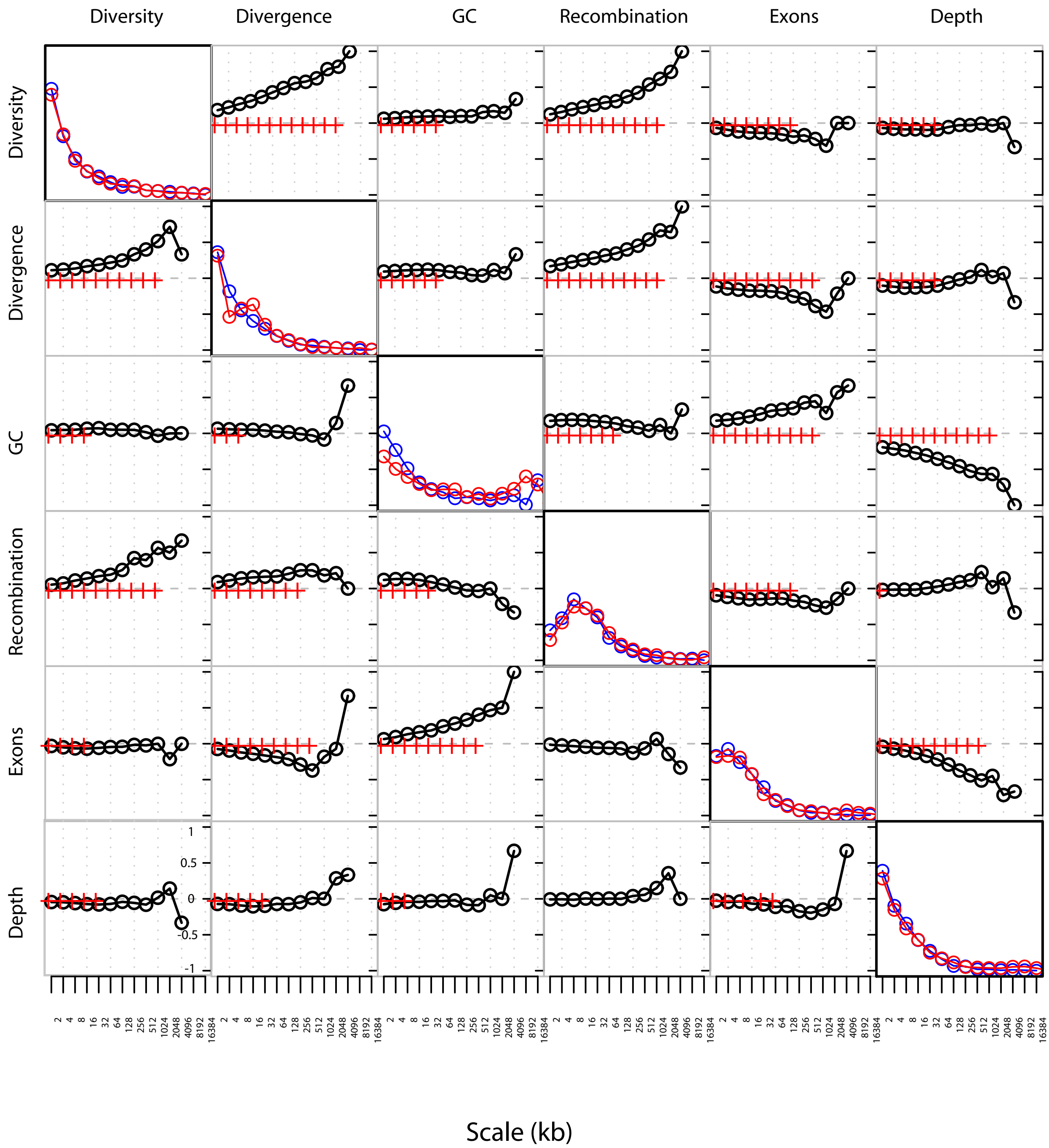

Spencer et al: Figure S1
