# Supplementary material for: The Influence of Recombination on Human Genetic Diversity
Source: PLoS Genet. 2006 Sep 22;2(9):e148. doi: 10.1371/journal.pgen.0020148 (PMC1575889; doi:10.1371/journal.pgen.0020148)
Supplement: Supplementary file 4 [file pgen.0020148.sd003.doc]

**The influence of recombination on human genetic diversity**

^1^Chris Spencer*, ^2^Panos Deloukas*, ^2^Sarah Hunt, ^3^Jim Mullikin, ^1^Simon Myers, ^1^Bernard Silverman, ^1^Peter Donnelly, ^4^David Bentley^†^ and ^1^Gil McVean^†^

^1^Department of Statistics, University of Oxford, UK.

^2^Wellcome Trust Sanger Institute, Hinxton, UK.

^3^ National Human Genome Research Institute, National Institutes of Health, Bethesda, MD, USA.

^4^Solexa Ltd, UK.

**SNP discovery and estimation of diversity**

Details on the polymorphisms identified (in build 34 coordinates) are available from

[www.stats.ox.ac.uk/~mcvean/C20/chr20.SNPdiscoveryInfo.gz](http://www.stats.ox.ac.uk/~mcvean/C20/chr20.SNPdiscoveryInfo.gz)

The file details the number of times each allele of a SNP (either the reference sequence, .ref or an alternative allele, .var) was identified from the collection of DNA samples used in SNP ascertainment. Details of each DNA are as follows

**CHIMP**: Mostly "Clint". Not used for SNP discovery, just for double hit counts. If the base was polymorphic in chimp, both alleles were set to zero. If ".var" is 1 for chimp, it's not guaranteed that the variant allele agrees with the variant allele in human. This disagreement probably happens less than 2% of the time.

**Cor10470**: Coriell cell line (Pygmy). See <http://locus.umdnj.edu/nigms/nigms_cgi/display.cgi?GM10470>

**Cor11321**: Coriell cell line (Chinese). See <http://locus.umdnj.edu/nigms/nigms_cgi/sample.cgi?CHINESE>
**Cor17109**: Coriell cell line (African American). See

<http://locus.umdnj.edu/nigms/nigms_cgi/sample.cgi?HD100AA>

**Cor17119**: Coriell cell line (African American). See <http://locus.umdnj.edu/nigms/nigms_cgi/sample.cgi?HD50AA>

**Cor7340**: Coriell cell line (CEPH from Utah). See <http://locus.umdnj.edu/nigms/nigms_cgi/sample.cgi?MOR50002>

**HuAA, HuCC, HuDD and HuFF**: Individuals from the Celera Human genome sequencing project. More information can be found in Science 291: 1304 (2001).

**G248 and NA15510**: Sequences from fosmid ends. See

<http://locus.umdnj.edu/nigms/nigms_cgi/sample.cgi?SEQVAR>
**BCMWGS_S213**: The SNP reads are from a pool of 8 unrelated adult African Americans, 4 male and 4 female enrolled in Houston, TX. The pool does not have a name. The 8 samples were derived from the Baylor Polymorphism Resource which includes >500 ethnically diverse samples.

**NIH24**: This is from an NIH SNP discovery panel of 24 ethnically diverse individuals, which was used by TSC for SNP discovery in a pooled form.

**WGSA**: This is a "mosaic" single haploid, i.e. the Celera assembly, as submitted to genbank under accession AADD00000000. See <http://www.ncbi.nlm.nih.gov/entrez/viewer.fcgi?db=nucleotide&val=42795668>

**CLONE**: All human clones from genbank compared to the reference genome sequence.

**EST**: All EST sequence compared to the reference sequence. Not used for SNP discovery, just used for double hit totals.

Note. If the DNA source was from a single individual, the "total" for either .ref or .var was only allowed to be either 0 or 1. For pools, the numbers of each allele seen in the alignment is reported.

Three summaries of diversity were calculated: (note that values are reported x10). Values for Pi were used in all analyses as a summary of local genetic diversity.

**Theta.** A version of Watterson’s estimate of theta, with a per base pair correction for read depth. For a window of *L* nucleotides with *S* segregating sites, where the read depth at base *i* is *k_i_*

$\hat{\theta}_{W}=S/{\frac{1}{L}}\sum_{i=1}^{L} \sum_{j=1}^{k_{i}} 1/j$

**Pi**. Average pairwise differences between those portions of read that map to the window, normalised for read length. If reads *i* and *j* both map to the window and have overlap *L_ij_* within the window and *k_ij_* differences within that overlap, the estimator is

$\pi=\sum_{i,j;i\neq j} k_{\text{ij}}L/{L_{\text{ij}}}$

**Heterozygosity**. For each SNP the minor allele frequency is estimated from the reads and heterozygosity is calculated as

$H=\sum_{i=1}^{L} 2\hat{f}_{i}(1-\hat{f}_{i})$

Note that under a neutral model both Theta and Pi should be unbiased estimators of the population mutation rate 4*N_e_u*, where *N_e_* is the effective population size and *u* is the per site per generation mutation rate.
