# Supplementary material for: The Influence of Recombination on Human Genetic Diversity
Source: PLoS Genet. 2006 Sep 22;2(9):e148. doi: 10.1371/journal.pgen.0020148 (PMC1575889; doi:10.1371/journal.pgen.0020148)
Supplement: Supplementary file 5 [file pgen.0020148.sd004.doc]

**The influence of recombination on human genetic diversity**

^1^Chris Spencer*, ^2^Panos Deloukas*, ^2^Sarah Hunt, ^3^Jim Mullikin, ^1^Simon Myers, ^1^Bernard Silverman, ^1^Peter Donnelly, ^4^David Bentley^†^ and ^1^Gil McVean^†^

^1^Department of Statistics, University of Oxford, UK.

^2^Wellcome Trust Sanger Institute, Hinxton, UK.

^3^ National Human Genome Research Institute, National Institutes of Health, Bethesda, MD, USA.

^4^Solexa Ltd, UK.

**Note on the relationship between correlation coefficients between raw and wavelet-transformed signals**

Suppose we have two signals, $x=\left\{ x_{1},x_{2},\ldots,x_{n} \right\}$ and $y=\left\{ y_{1},y_{2},\ldots,y_{n} \right\}$, where *n* is a power of two; $n=2^{k}$. We perform a discrete wavelet transformation of the signals to obtain a series of detail coefficients at each scale; $W^{\lambda}(x)=$ and $W^{\lambda}(y)=$, where $\lambda$ is the scale, $\lambda\in\left\{ 1,2,\ldots,k \right\}$, $m_{\lambda}$is the number of detail coefficients at scale $\lambda$. It can be shown that the covariance between the original signals can be rewritten in terms of the detailed coefficients at each scale.

$\begin{aligned} \text{Cov}(x,y)=\frac{1}{n}\sum_{i=1}^{n} x_{i}y_{i}-\left( \frac{1}{n}\sum_{i=1}^{n} x_{i} \right)\left( \frac{1}{n}\sum_{i=1}^{n} y_{i} \right) \\ \frac{1}{n}\sum_{\lambda=1}^{k} \sum_{i=1}^{m_{\lambda}} w_{\text{xi}}^{\lambda}w_{\text{yi}}^{\lambda} \end{aligned}$

By rewriting the various quantities it follows that

$\begin{aligned} \text{Cor}(x,y)=\frac{\text{Cov}(x,y)}{\sqrt{\text{Var}(x)\text{Var}(y)}} \\ =\sum_{\lambda=1}^{k} (\alpha_{x}^{\lambda}\alpha_{y}^{\lambda})^{\frac{1}{2}}\rho(w_{x}^{\lambda},w_{y}^{\lambda}) \end{aligned}$

Where

$\alpha_{x}^{\lambda}=\frac{\sum_{i=1}^{m_{\lambda}} \left( w_{\text{xi}}^{\lambda} \right)^{2}}{\text{Var}(x)}\text{ , and }\alpha_{y}^{\lambda}=\frac{\sum_{i=1}^{m_{\lambda}} \left( w_{\text{yi}}^{\lambda} \right)^{2}}{\text{Var}(y)}$

and

$\rho(w_{x}^{\lambda},w_{y}^{\lambda})=\frac{\sum_{i=1}^{m_{\lambda}} w_{\text{xi}}^{\lambda}w_{\text{yi}}^{\lambda}}{\sqrt{\left( \sum_{i=1}^{m_{\lambda}} \left( w_{\text{xi}}^{\lambda} \right)^{2} \right)\left( \sum_{i=1}^{m_{\lambda}} \left( w_{\text{yi}}^{\lambda} \right)^{2} \right)}}$

Note that $\rho^{2}(w_{x}^{\lambda},w_{y}^{\lambda})$ is the proportion of the variance in $w_{y}^{\lambda}$ explained by a linear model with predictor $w_{x}^{\lambda}$ in which the intercept is zero. Alternatively, if the detail coefficients at each scale have zero mean then $\rho(w_{x}^{\lambda},w_{y}^{\lambda})$ is the Pearson correlation coefficient between the detail coefficients of *x* and *y* at scale *λ*. Consequently, the Pearson correlation coefficient between the original signals is approximately a weighted sum over scales of the correlation between the detail coefficients at a given scale, where the weight is the geometric average of the proportion of the variance in explained by that scale for the two signals. For all genomic features considered here there mean of the detailed coefficients at each scale is always close to zero.
